# Low-Temperature Sintering Mechanism and Bonding Performance of Submicron Triangular Silver Paste for Advanced Electronic Packaging

**DOI:** 10.3390/ma19112182

**Published:** 2026-05-22

**Authors:** Pengrong Lin, Zirui Tong, Shimeng Xu, Yiping Wang, Shang Wang, Yanhong Tian

**Affiliations:** 1 Beijing Microelectronics Technology Institute, Beijing 100076, China; lpr666@vip.163.com (P.L.); buaa_xsm@163.com (S.X.); 2 Department of Mechanical Engineering, Tsinghua University, Beijing 100084, China; 3 State Key Laboratory of Precision Welding & Joining of Materials and Structures, Harbin Institute of Technology, Harbin 150001, China; 25b909045@stu.hit.edu.cn (Z.T.); 22b309012@stu.hit.edu.cn (Y.W.); tianyh@hit.edu.cn (Y.T.)

**Keywords:** paste, silver flakes, sintering mechanism, advanced electronic packaging

## Abstract

**Highlights:**

- We characterize a submicron triangular silver flake paste with high strength and low porosity at a low temperature.
- The submicron silver flakes have two different sintering stages.
- The sharp corners and edges of the submicron triangular silver flakes are easier to sinter than the surface.

**Abstract:**

Silver pastes are promising joining materials for use in advanced electronic packaging, particularly in power electronics, due to their capacity for low-temperature joining and high-temperature operation. However, spherical silver particles suffer from low sintering efficiency and high porosity due to their limited contact areas. In this study, a submicron triangular silver flake paste was investigated to overcome these limitations. A high joint strength of 35.2 MPa with a low porosity of 9.48% was achieved at 250 °C via pressureless sintering. A relatively high thermal conductivity (134.9 W/(m·K)) was also achieved. This enhanced sintering performance is attributed to a unique mechanism involving distinct same-layer and inter-layer sintering processes at different temperatures. With these findings, we demonstrate that the submicron triangular silver flake paste is a high-strength, high-thermal-conductivity, low-temperature bonding material for advanced electronic packaging.

## 1. Introduction

Third-generation wide-bandgap semiconductors, such as silicon carbide (SiC) and gallium nitride (GaN), are promising candidates for use in power device chip materials due to their wide bandgap, high breakdown voltage, excellent thermal stability, and high carrier mobility [1,2,3,4,5]. Compared to Si-based devices, SiC power devices can withstand peak temperatures above 600 °C for a short period of time under extreme conditions, greatly improving the conversion efficiency [6]. However, the advanced packaging of these third-generation semiconductors poses challenges for the processing and reliability of packaging materials. Taking electric vehicles as an example, the onboard module may operate in a high-temperature environment exceeding 100 °C for a long time, and extreme junction temperature can reach 175 °C [7]. In the foreseeable future, with the development of power devices, the working temperature will further increase, leading to more stringent requirements for the heat dissipation and high-temperature reliability of packaging materials.

Conventional Sn-based solders and indium cannot meet high-temperature requirements because their melting points are usually below 300 °C. At elevated temperatures, the solder joint performance deteriorates rapidly, seriously affecting the reliability of the device [8,9]. Silver nanopastes with low sintering temperatures have emerged as the material of choice for SiC power electronics owing to their high thermal conductivity, low sintering pressure requirements, and excellent high-temperature performance [10,11]. Adding coefficient of thermal expansion (CTE)-compatible fillers can effectively improve the reliability of the silver nanopaste joint [12]. However, silver nanoparticles agglomerate during storage and sintering, resulting in high-porosity joints with poor thermal and electrical conductivities; they are also typically coated with organic solvents during the synthesis process. Therefore, the sintering of silver nanopastes often requires harsh conditions such as high temperature (about 250 °C) or high pressure (about 10 MPa). Micro/nano-silver paste, which contains large particle skeletons and small particle fillers, can significantly reduce joint porosity, but it also lowers sintering efficiency [13,14]. To overcome these drawbacks, a submicron silver flake paste was developed, improving sintering efficiency and reducing joint porosity via flake self-assembly. However, the sintering mechanism of the flakes remains unclear.

In this study, the submicron triangular silver flakes were systematically investigated to elucidate the fracture mechanisms and microstructural evolution of the resulting joints. The unique sintering behavior of these flakes was analyzed under different temperatures, and high-strength, high-thermal-conductivity, low-porosity silver joints were achieved under optimized sintering conditions, demonstrating their potential for high-temperature electronic packaging applications.

## 2. Materials and Methods

### 2.1. Materials

The submicron silver flake paste was supplied by Kyocera Corporation (Kyoto, Japan). Cu substrates (1 mm × 1 mm, electroless Ni/immersion Au) were provided by Shenzhen JLC Technology Group Co., Ltd.(Shenzhen, China). A nickel layer and a gold layer were deposited on the copper via Electroless Nickel/Immersion Gold (ENIG) plating.

### 2.2. Fabrication Process

**Figure 1:**
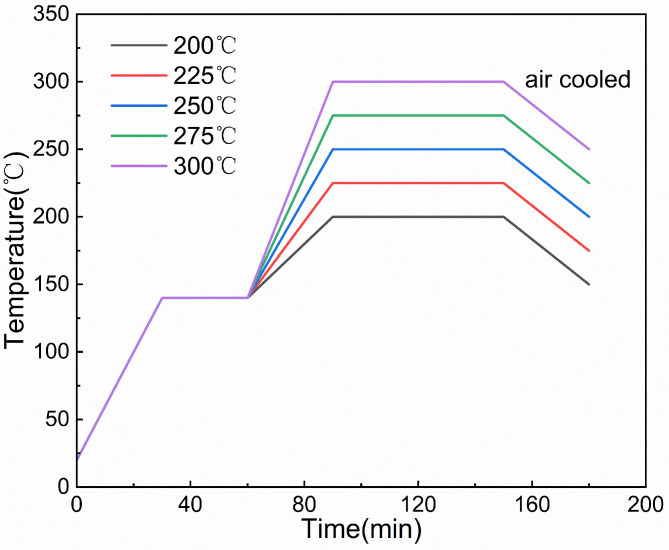
The reflow profile for the silver paste sintering process.

In this study, we employed two primary sintering process flows: sandwich-like solder joint fabrication and paste sintering analysis. The specific steps taken were as follows:

Solder joint fabrication: Solder joints with a sandwich structure were fabricated using the aforementioned silver paste and ENIG-finished copper substrates. The copper substrates were prepared as 1 mm × 1 mm squares and 6 mm diameter disks. The fabrication process was as follows: The gold-plated surfaces of the copper pieces were lightly polished with 5000-grit sandpaper. A 100 μm thick screen was used to stencil-print a uniform layer of the silver paste onto the center of the disc. The square piece was then carefully aligned and placed on top of the printed silver paste. A slight pressure was applied to ensure complete contact. Finally, the assembled sandwich structure was placed in the center of the tube furnace. Sintering was performed according to the heating profile shown in Figure 1 with a N_2_ atmosphere. In the sintering curve, the sample was heated from 20 °C to 140 °C at a rate of 4 °C/min, held at that temperature for 30 min, and then further heated to the peak temperature over the next 30 min. After being held at the peak temperature for 60 min, the sample was allowed to cool naturally to room temperature. The resulting solder joints were used for subsequent cross-sectional analysis and strength testing.

Paste sintering procedure: To study the sintering behavior of the submicron triangular silver flakes, the following procedure was employed. The as-received silver paste was diluted with appropriate amounts of ethanol and acetone. The mixture was ultrasonicated and then centrifuged to isolate the submicron triangular silver flakes. The separated flakes were uniformly re-dispersed in ethanol to form a suspension with a concentration of 10 mg/mL. Subsequently, 20 µL of this suspension was drop-casted onto a clean silicon wafer. The samples were then sintered on a hot plate at temperatures of 215 °C, 230 °C, and 250 °C for durations of 30 s, 5 min, and 10 min, respectively. The sintered samples prepared under these varied time–temperature conditions were used to analyze the sintering process of the submicron triangular silver flakes.

### 2.3. Characterization

X-ray Diffraction (XRD) of the submicron triangular silver flakes was conducted using X’Pert PRO MPD (PANalytical B.V., Almelo, The Netherlands). The morphologies of the cross-section and the sintered silver paste were investigated using a HITACHI SU8220 scanning electron microscope (SEM) (HITACHI, Tokyo, Japan). The microscopic morphology and defect characterization of the submicron triangular silver flakes were investigated using a Talos F200S transmission electron microscope (Thermo Fisher Scientific, Waltham, MA, USA). The tube furnace (Hefei Kejing Material Technology Co., Ltd., Hefei, China) was used to reflow soldering. The thermal change in the submicron triangular silver flakes was analyzed using a 404 F3 Pegasus differential scanning calorimeter (DSC) (NETZSCH, Munich, Germany). Shear testing was performed using a Dage S4000 IC packaging bond tester (Dage, Aylesbury, UK). The thermal conductivity of the silver paste was analyzed via an LFA 467 HyperFlash thermal conductivity tester (NETZSCH, Munich, Germany).

### 2.4. Porosity Statistical Method

The porosity of solder joints was statistically analyzed using ImageJ software (Version 1.47). Typically, in such experiments, three sets of samples are prepared under the same conditions. After grinding and polishing, three scanning images are selected from each sample for porosity calculation, and the average value is taken. First, we selected a scanned image of the interior of a silver sintered body and used the ImageJ-Threshold module to process the image. The porous parts of the solder joints were displayed in real time. The obtained data represented the porosity of the solder joints. We then repeated the operation with multiple scanned images of solder joints under the same parameter, and the processed data represent the average porosity of the solder joints under that parameter.

## 3. Results

### 3.1. The Morphology of Submicron Silver Flakes

**Figure 2:**
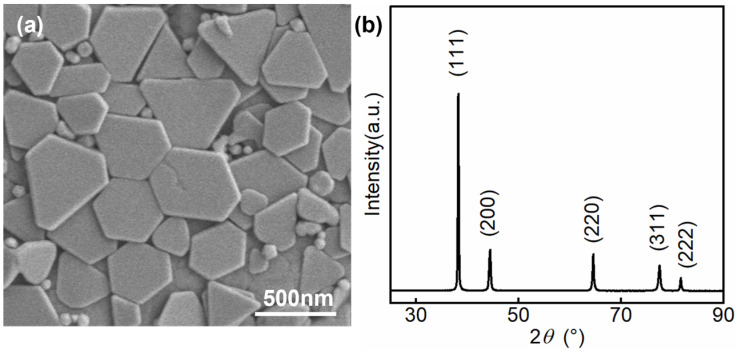
(**a**) The SEM image of the submicron silver flakes. (**b**) The XRD pattern of the submicron silver flakes.

The different microstructures of the micro/nano-silver particles certainly influence the sintering process of the silver pastes [15]. Figure 2a shows the morphology of the submicron silver flakes. Most silver flakes were hexagonal in shape, with shorter sides on three sides, forming an approximate triangle. The flakes exhibited specific orientation relationships between their surfaces and side facets. Regardless of whether the silver flakes were approximately triangular or hexagonal, their crystal structure and facet orientation relationships remained the same. Hence, the silver flakes in this study will be referred to as ‘submicron triangular silver flakes’. Their formation, as described by Xia et al. [16], involved a layer-by-layer edge-growth mechanism during slow nucleation, resulting in multi-layer stacks separated by stacking faults. During the growth process, each crystal plane had a specific orientation relationship. Moreover, compared with the standard lattice, the XRD pattern (Figure 2b) indicated a pronounced <111> orientation, suggesting that the silver flake was predominantly composed of (111) facets.

**Figure 3:**
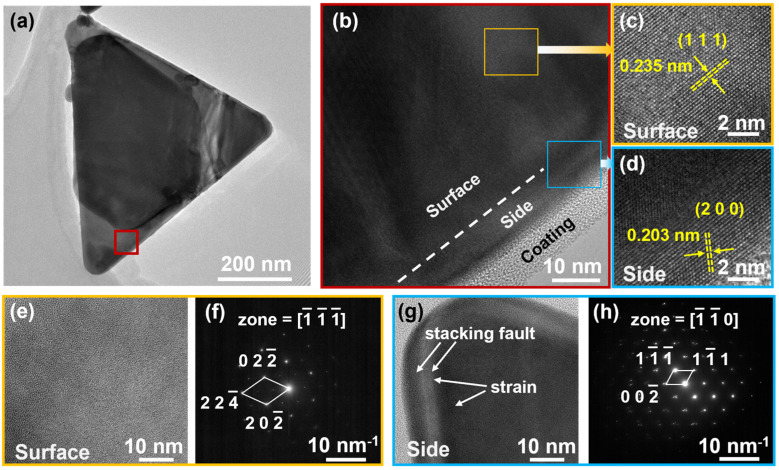
(**a**–**d**) The TEM and HRTEM images of the silver flakes. (**e**) The HRTEM and (**f**) electron diffraction of the surface of the submicron silver flakes. (**g**) The HRTEM and (**h**) electron diffraction of the side of the silver flakes.

To clarify the surface crystallographic correlation and defect distribution of the submicron triangular silver flakes, TEM analysis was performed. The TEM image in Figure 3b shows the overall morphology of the submicron triangular silver flakes and the microstructure at the edges. The edge length of the silver flake is about 500 nm, and the size and morphology of the silver flakes are relatively uniform, with smooth edges at the sharp corners. The side edge exhibited significant defect enrichment, as inferred from the lower interplanar spacing of the (111) plane and the atomic misalignment identified at the boundary. The silver flake contains two parallel stacking faults between the upper and lower crystal layers [8], and some stacking fault boundaries can also be observed in Figure 3c. The high-resolution and electron diffraction analysis depicted in Figure 3e–h shows that a stress concentration exists at the edges of the flakes, with multiple stacking faults distributed along the upper and lower edges of the flakes.

### 3.2. The Sintering Mechanism of Submicron Silver Flakes

**Figure 4:**
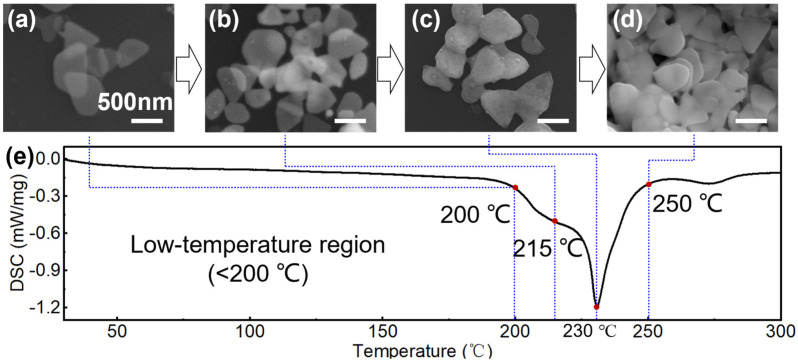
(**a**–**d**) SEM morphologies of sintered silver flakes at 200 °C, 215 °C, 230 °C and 250 °C for 30 s. The scale bar of the SEM images is 500 nm. (**e**) The corresponding DSC curve.

The sintering behavior of the submicron triangular silver flakes was characterized by DSC, as shown in Figure 4. The onset of sintering was observed at around 200 °C (Figure 4a), with the DSC curve presenting an energy release trend, but the morphology of the silver flake did not change, maintaining a triangular structure. The first significant change was observed from 215 °C to 230 °C. There was a noticeable melting of the edge for some small silver flakes when the temperature increased to near 215 °C. Sintering necks were observed at various junctions, including edge-to-edge and edge-to-corner types (Figure 4b). The first exothermic stage observed in the DSC curve corresponded to same-layer sintering. At 230 °C, the connections between same-layer submicron silver flakes were observed (Figure 4c). The boundaries between the flake layers started to close, and the sintered structure transformed to a more continuous structure. This suggests that the second exothermic peak observed in the DSC curve corresponded to inter-layer sintering. When the temperature increased to 250 °C, the distinct morphology of the submicron silver flakes disappeared, becoming a tight sintered network (Figure 4d). Meanwhile, the sintering process was strongly correlated with temperature, while time had little influence. The DSC profile stabilized under continuous heating, indicating that silver flakes complete two-stage sintering between 230 °C and 250 °C (Figure 4e).

**Figure 5:**
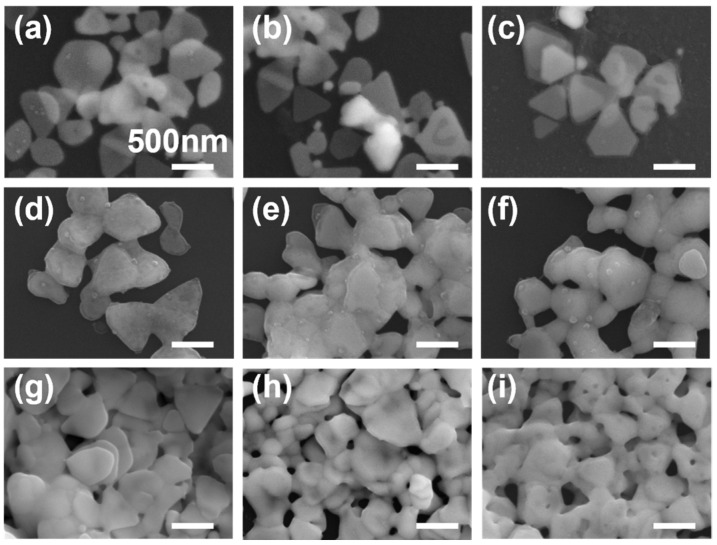
(**a**–**c**) SEM morphologies of sintered silver flakes at 215 °C, 230 °C and 250 °C for 30 s, (**d**–**f**) 5 min, and (**g**–**i**) 10 min, respectively, in N_2_. The scale bar of the SEM images is 500 nm.

To isolate the effect of sintering time, a series of quasi in situ sintering experiments were performed on the submicron triangular silver flakes. Temperatures of 215 °C, 230 °C, and 250 °C were selected with heating durations of 30 s, 5 min, and 10 min, as shown in Figure 5. At 215 °C, the sintering mechanism of the submicron triangular silver flakes was dominated by same-layer sintering, including edge-to-edge and edge-to-corner types. Increasing the sintering time did not change the sintering mechanism. When the temperature increased to 230 °C, significant connections between same-layer submicron silver flakes were observed within just 30 s. With prolonged sintering, the boundaries between the flake layers started to close, and the sintered structure transformed to a more continuous structure. The sintering mechanism tended to involve the simultaneous progression of both same-layer sintering and inter-layer sintering. At 250 °C, silver flakes tended to generate a complete sintered network. As the time increased, the morphological characteristics of the submicron triangular silver flakes gradually disappeared. The network pores gradually closed and spheroidized as the silver element lattice received migrated silver atoms.

The unique sintering mechanism of the submicron triangular silver flakes is theoretically explained as follows: The analysis employed the two-sphere sintering model for silver nanoparticles and systematically considered the contributions of different diffusion mechanisms [17,18,19,20]. Consequently, the atomic flux at the sintering neck equals the algebraic sum of the fluxes of each mechanism multiplied by their respective cross-sectional areas. The flux of each diffusion type is related to its diffusion coefficient, which can generally be described by an Arrhenius-type equation [21]. Since micro/nano-particles inherently contain high-density defects both on the surface and within the bulk, introduced during preparation and sintering, vacancy diffusion and dislocation diffusion were incorporated in addition to the conventional grain boundary and surface diffusion mechanisms. Given the significant size effects and defect distributions at the edges and corners of the submicron triangular silver flakes, this mechanism is also applicable to the submicron silver flakes described in this study. The total effective diffusion coefficient is defined in Equation (1):

(1) $$D_{total}=D_{v}+\rho_{d}D_{d}+\delta_{gb}D_{gb}+\delta_{s}D_{s}$$

In the equation, $D_{total}$ is the total effective diffusion coefficient, $D_{v}$ is the vacancy diffusion coefficient, $\rho_{d}$ is the dislocation density, $D_{d}$ is the dislocation pipe diffusion coefficient, $\delta_{gb}$ is the grain boundary width, $D_{gb}$ is the grain boundary diffusion coefficient, $\delta_{s}$ is the surface layer thickness, and $D_{s}$ is the surface diffusion coefficient.

Based on the characterization of the submicron triangular silver flakes mentioned above, we can conclude that the defect density at the corners and edges of the submicron triangular silver flakes is significantly higher than that on the surface. The HRTEM images of the flake edges and corners revealed distinct stacking faults and vacancy distributions. The electron diffraction patterns displayed multiple sets of diffraction spots, indicating the presence of the strain field and multiple stacking faults in these regions. In contrast, the results for the flake surfaces were markedly different, showing ordered atomic arrangements with no obvious defects. For nanoparticle sintering, surface and grain boundary diffusion play dominant roles. The size effect, coupled with synergistic multi-path diffusion, significantly enhances atomic diffusion rates at the sintering necks. In the triangular submicron silver flake system, the curvature radius at the sharp corners of the flakes is particularly low, making the size effect even more pronounced. Therefore, sintering neck formation temperature at the edges/corners of submicron triangular silver flakes is relatively close to that of silver nanoparticles. Moreover, the aforementioned experimental findings confirm a higher defect density at the edges and corners, substantially increasing the overall diffusion coefficient. In summary, based on the qualitative analysis of the diffusion coefficient formula, the diffusion coefficient is greater at the edges and corners than at the surface. Therefore, at a lower temperature (215 °C), the sintering mechanism was mainly based on same-layer sintering, manifested as edge-to-edge and edge-to-corner interconnection. The inter-layer sintering dominated by surface sintering required a higher temperature (230 °C).

### 3.3. The Characterization of Solder Joint Morphology and Performance

**Figure 6:**
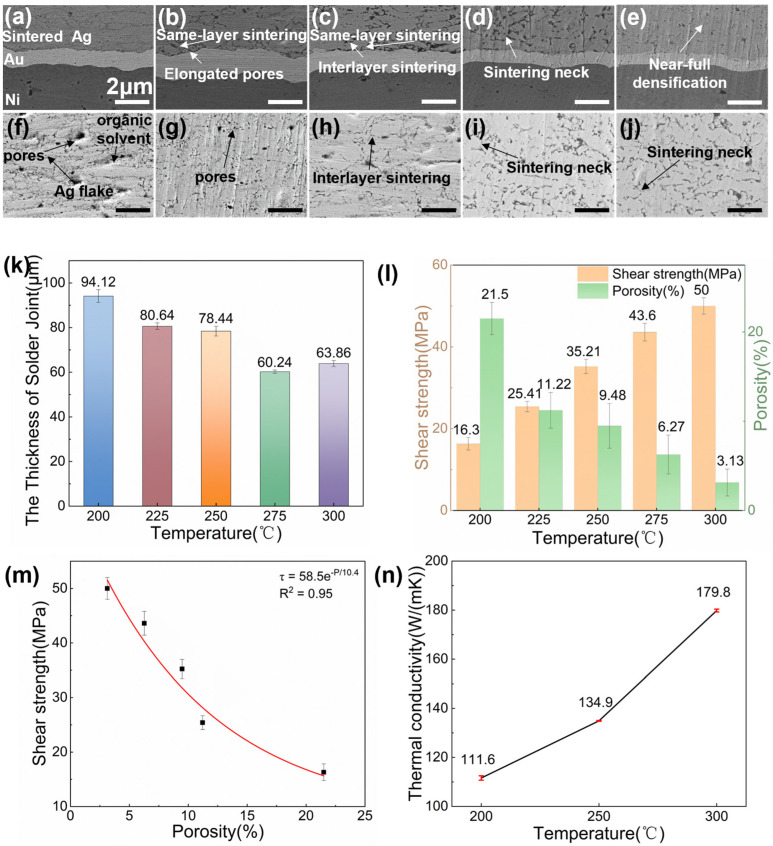
(**a**–**e**) The cross-section and (**f**–**j**) sintered silver paste morphology of the sandwich-like structure at 200 °C, 225 °C, 250 °C, 275 °C, and 300 °C. The scale bar of the SEM images is 2 μm. (**k**) The thickness of the pressureless sintered solder joints at different temperatures. (**l**) The shear strength and the porosity of the sandwich-like structure. (**m**) The quantitative relationship between solder joint porosity and shear strength, includes scatter plot and fitting curve (Red line). (**n**) The thermal conductivity of the silver paste sintered at 200 °C, 250 °C, and 300 °C.

To confirm this hypothesis, a series of sintering tests were conducted. The cross-section and sintered silver paste morphology are shown in Figure 6. The thickness of the as-printed silver paste layer prior to sintering was approximately 100 µm. The variation in joint thickness after sintering at different peak temperatures is shown in Figure 6k. The average joint thickness gradually decreased with increasing peak sintering temperature and eventually stabilized at around 60 µm. This can be attributed to the following mechanisms: In the initial state, the submicron triangular silver flakes were randomly oriented and distributed within the organic vehicle. As the organic components volatilized and the flakes underwent reorientation during sintering, the average joint thickness decreased significantly, a phenomenon particularly noticeable at 250 °C and lower temperatures. With a further temperature increase, sintering between the flakes progressed towards completion. The enhanced atomic diffusion led to a substantial reduction in internal pores within the joint and vacancy density within the flakes, resulting in a further decrease in thickness. At 300 °C, the joints were considered fully sintered, and the average thickness remained essentially stable. The change in solder joint thickness also affected the strength of the solder joint and the porosity of the sintered body. Subsequently, the cross-sectional morphology of the solder joints was examined. At lower sintering temperatures (200 °C), a large number of unsintered submicron flakes were observed in the joint, though the sintering quality of the solder joints was poor. When the temperature increased to 225 °C, the porosity inside the solder joint gradually decreased, declining from an initial value of 21.5% to 10%. This is because higher temperatures promoted the sintering of same-layer flakes to a layer of sintering network. As the sintering temperature increased above 250 °C, the pores gradually evolved from their previous irregular shape to have a circular and droplet shape due to the higher surface energy of the irregular pores, which attracted atomic diffusion. At 300 °C, as the sintering temperature increased, the interfacial contact area increased due to silver atom diffusion and the porosity steadily decreased to 3.13%.

The relationship between solder joint porosity and shear strength was also quantitatively analyzed. The porosity values at different temperatures represent the average measured from experiments, while the shear strength data include all experimental measurements with error bars, as shown in Figure 6m. To fit the experimental data, we extensively reviewed the relevant literature. Research related to nano-silver sintering primarily focuses on constructing constitutive mechanical equations for sintered bodies, which is not directly applicable to the mechanical performance analysis of the sandwich solder joints that we performed in this study [22,23,24]. Therefore, the sandwich-like solder joints were regarded as brittle porous materials, and we employed the classical exponential decay model for fitting, as expressed in Equation (2). This model, known for its simplicity, is widely used for predicting the shear strength of brittle materials such as ceramics and concrete:

(2) $$\tau =\tau_{0}e^{-bP}$$

In the equation, τ is the measured shear strength, $\tau_{0}$ is the theoretical intrinsic strength of the material, P is the porosity, and b is an empirical constant. The fitting result was R^2^ = 0.95, indicating a good fit. It should be noted that the shear strength of sintered silver paste joints is strongly dependent on sintering parameters such as temperature, time, and pressure, all of which influence the accuracy of the fitted equation.

As a packaging material for third-generation semiconductors, thermal conductivity is a crucial criterion for evaluating solder joint performance. Silver paste sintered bodies were prepared at 200 °C, 250 °C, and 300 °C, and their thermal conductivity was measured using the laser flash method. As the sintering temperature increased, the thermal conductivity of the sintered bodies gradually increased, reaching 134.9 W/(m·K) at 250 °C. This excellent heat transfer performance ensures its suitability for third-generation semiconductor packaging.

**Figure 7:**
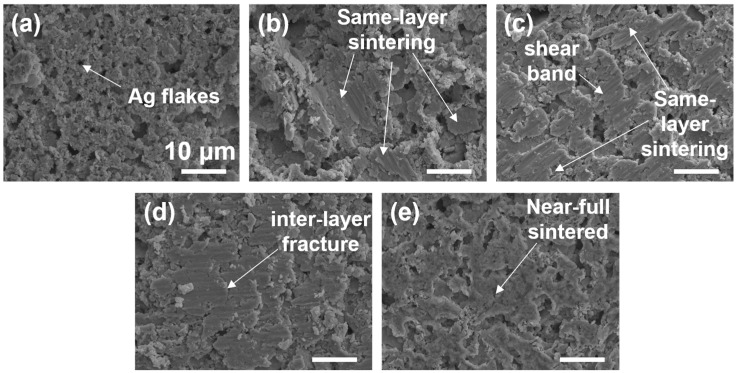
(**a**–**e**) The fracture morphologies of joints sintered at 200 °C, 225 °C, 250 °C, 275 °C, and 300 °C, respectively.

The porosity of the joining will influence its strength and failure mode. Hence, the fracture morphology and shear strength were correlated. As shown in Figure 7, all fracture sites were located within the sintered joints, indicating good bonding between the silver paste and the gold layer. In general, as the sintering temperature increases, the submicron triangular silver flakes progressively sinter toward completion. At 200 °C, as shown in Figure 7a, flakes oriented in various random directions are clearly visible on the fracture surface, indicating that sintering has not yet begun at this temperature, and individual flake morphology remains distinct. When the sintering temperature reaches 225 °C, same-layer sintering between flakes begins, while inter-layer sintering has not yet commenced. Consequently, the fracture surface shows agglomerated and coarsened flakes alongside a significant number of isolated individual flakes. Upon heating to 230 °C, same-layer sintering nears completion, and inter-layer sintering initiates. The fracture surface exhibits numerous fully coalesced flake aggregates, and the extent of inter-layer sintering becomes the dominant factor determining fracture morphology. At 250 °C, the joint formed via inter-layer sintering, though it was not fully completed, as shown in Figure 7c. At 275 °C, all fracture sites were located within the sintered joints, indicating good bonding between the silver paste and the gold layer. Cracks and pores between the silver flake layers became the primary stress concentration sites. Increasing the sintering temperature to promote inter-layer sintering alleviated such stress concentrations, thereby significantly enhancing the mechanical properties of the solder joints. At 300 °C, silver flakes were fully sintered, and the fracture in interface zone showed brittleness rupture.

Overall, the fracture mechanism of the solder joints is strongly correlated with the sintering process of the submicron triangular silver flakes. Same-layer sintering initiates at a lower temperature and proceeds more readily to completion. Prior to the onset of same-layer sintering, joint strength is extremely low, relying primarily on bonding between the flakes and the gold pad. Following same-layer sintering, joint strength increases substantially. At the characteristic temperature of 215 °C, the fracture surface from same-layer sintering appears distinctly inclined. This is likely due to the fact that during flake rearrangement, the surfaces of inter-layer particles become tilted at an angle relative to the pad. These multi-angled, inter-layer sintered flakes collectively sustain joint strength. At this stage, stress concentrates at the pores between different sets of inter-layer sintered flake clusters. Increasing the sintering temperature to promote inter-layer sintering is essentially a method to alleviate such stress concentrations. As temperature rises, inter-layer particle sintering completes while inter-layer sintering progressively strengthens, significantly enhancing the overall joint strength. However, as previously described, the defect density on the flake surfaces is much lower than at edges and corners, and the size effect is substantially diminished. This results in a higher activation temperature for inter-layer sintering and inferior sintering efficacy at comparable temperatures. Although the observed overall porosity decreases, the fact that stress concentrates at the weak bonding interfaces between flake layers remains unchanged. Therefore, joint fracture almost exclusively occurs in the inter-layer sintered regions of the submicron triangular silver flakes.

## 4. Conclusions

In summary, the sintering performance and mechanism of the submicron triangular silver flake paste were analyzed. Through observing microstructural characterization and quasi in situ sintering, we discovered, for the first time, that the edges and corners of the silver flakes possess a high density of high-energy defects. As a result, a two-stage sintering process is identified. The first stage, dominated by same-layer sintering between edges and corners, occurs at 215 °C, followed by a second stage characterized by inter-layer sintering between the top and bottom surfaces at 230 °C. Due to this unique sintering mechanism, the silver flake pastes reached a strength of 35.2 MPa, a thermal conductivity of 134.9 W/(m·K), and a porosity of 9.48% at 250 °C. These high-strength and low-porosity joints sintered under low-temperature conditions were suitable for use in high-power device packaging.

## Data Availability

The original contributions presented in this study are included in the article. Further inquiries can be directed to the corresponding author.
